# Erythema Multiforme Following Vaccination in a 4-Month-Old Infant

**DOI:** 10.4269/ajtmh.25-0630

**Published:** 2026-03-26

**Authors:** Deepika Chauhan, Apoorva Sharma, Dipankar De

**Affiliations:** Department of Dermatology, Venereology and Leprology, Postgraduate Institute of Medical Education and Research, Chandigarh, India

## INTRODUCTION

Erythema multiforme (EM) is a hypersensitivity reaction characterized by target skin lesions, often triggered by infections (e.g., *Herpes simplex virus*) or medications.^1^ Although vaccines are rare culprits, isolated cases of EM after immunization have been reported in adults and children.^2^ We present a case of EM in a 4-month-old infant temporally linked to the administration of the pentavalent, rotavirus, and inactivated poliovirus (IPV) vaccines.

**Figure 1.**
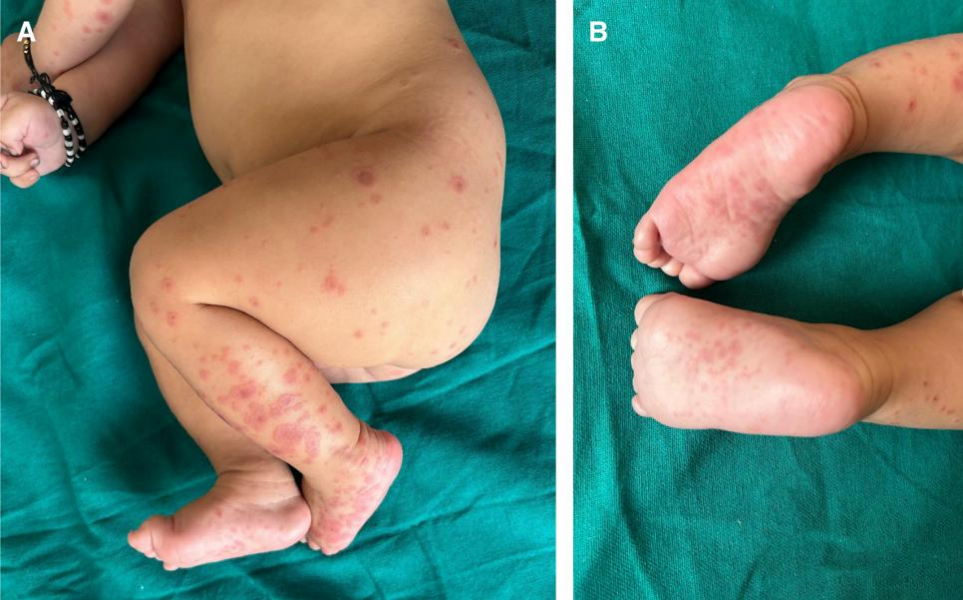
(**A**) Target lesions on the extremities of a 4-month-old infant diagnosed with erythema multiforme postvaccination. (**B**) Classic target lesions with three zones seen on the child’s soles.

A 4-month-old male presented with a 48-hour history of erythematous, nonpruritic, target lesions distributed symmetrically on his legs, arms, palms, and soles (Figure 1A and B). Two days before the presentation, he had received the third dose of the pentavalent (diphtheria, tetanus, pertussis, Hib, hepatitis B [DTaP-Hib-HepB]), rotavirus, and IPV vaccines. There was no history of fever, respiratory symptoms, or recent infections. The infant had no prior adverse reactions to vaccinations.

Cutaneous examination revealed multiple typical target lesions (<3 cm) with central duskiness and raised edges, consistent with EM minor.^3^ Mucosal surfaces were spared. Differential diagnoses of vasculitis and viral exanthems were excluded based on morphology and absence of systemic symptoms. The infant was diagnosed with vaccination-associated EM. The child was managed conservatively. Lesions resolved completely within seven days without scarring.

EM is uncommon in infants, and vaccine-associated cases are exceedingly rare. The temporal association (onset after 48 hours postvaccination) and absence of other triggers strongly suggested immunization as the cause. The pentavalent vaccine contains multiple antigens (DTaP-Hib-HepB) that may trigger a hypersensitivity reaction in susceptible individuals. The pathophysiology of EM involves immune complex deposition and cytotoxic T-cell activation, often triggered by antigens mimicking host proteins.^1^ Most vaccine-related cutaneous reactions are mild (e.g., local erythema). EM represents a type III hypersensitivity response.^4^ Huff et al. reported EM as a rare but documented adverse event following immunization, particularly with live-attenuated vaccines.^5^ However, inactivated vaccines like IPV and pentavalent formulations have also been implicated.^2^ This present case aligns with prior reports of EM following DTaP-containing vaccines. Notably, the infant’s rapid response to symptomatic management underscores the typically self-limiting nature of EM minor.^3^ Clinicians should consider vaccination history when evaluating infants with acute target eruptions. Although rare, EM can occur following routine infant vaccinations. Early recognition and conservative management often yield favorable outcomes. Further surveillance is needed to clarify the risk of EM associated with multiantigen vaccines.
